# (*E*)-1,1-Diphenyl-2-(thio­phen-2-yl­methyl­idene)hydrazine

**DOI:** 10.1107/S1600536813033874

**Published:** 2013-12-21

**Authors:** Blanca M. Cabrera-Vivas, Marcos Flores-Alamo, Ruth Meléndrez-Luévano, Lidia Meléndez-Balbuena, Juan C. Ramirez

**Affiliations:** a Facultad de Ciencias Químicas, Benemérita Universidad Autónoma de Puebla 72570, Puebla, Pue., Mexico; b Facultad de Química, Universidad Nacional Autónoma de México, 04510, México DF, Mexico

## Abstract

The asymmetric unit of the title compound, C_17_H_14_N_2_S, consists of two crystallographically independent mol­ecules with similar conformations. The dihedral angles between the phenyl rings are 89.32 (5) and 82.80 (5)° in the two mol­ecules. In the crystal, mol­ecules are linked by C—H⋯π inter­actions, forming a three-dimensional network.

## Related literature   

For biological applications of hydrazine derivatives, see: Vogel *et al.* (2008 ▶); Moreira *et al.* (2012 ▶); Vicini *et al.* (2009 ▶); Belkheiri *et al.* (2010 ▶); Shen *et al.* (2011 ▶).
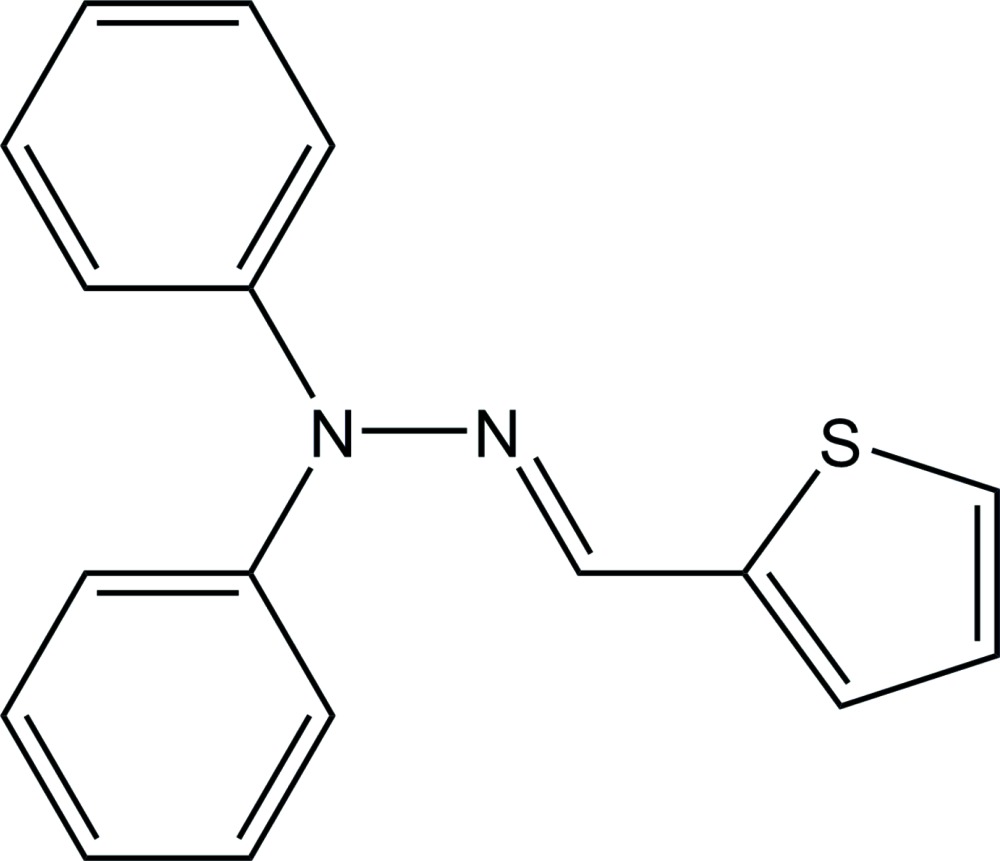


## Experimental   

### 

#### Crystal data  

C_17_H_14_N_2_S
*M* *_r_* = 278.36
Triclinic, 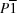

*a* = 9.8336 (11) Å
*b* = 9.8665 (9) Å
*c* = 16.6357 (8) Å
α = 100.290 (6)°
β = 101.696 (7)°
γ = 109.129 (9)°
*V* = 1439.9 (2) Å^3^
*Z* = 4
Mo *K*α radiation
μ = 0.22 mm^−1^
*T* = 144 K
0.58 × 0.51 × 0.36 mm

#### Data collection  

Agilent Xcalibur (Atlas, Gemini) diffractometer
Absorption correction: multi-scan (*CrysAlis RED*; Agilent, 2012 ▶) *T*_min_ = 0.916, *T*_max_ = 0.939
10771 measured reflections
5672 independent reflections
4856 reflections with *I* > 2σ(*I*)
*R*_int_ = 0.020

#### Refinement  

*R*[*F* ^2^ > 2σ(*F* ^2^)] = 0.039
*wR*(*F* ^2^) = 0.099
*S* = 1.07
5672 reflections
361 parameters
H-atom parameters constrained
Δρ_max_ = 0.23 e Å^−3^
Δρ_min_ = −0.33 e Å^−3^

### 

Data collection: *CrysAlis PRO* (Agilent, 2012 ▶); cell refinement: *CrysAlis PRO*; data reduction: *CrysAlis RED* (Agilent, 2012 ▶); program(s) used to solve structure: *SHELXS97* (Sheldrick, 2008 ▶); program(s) used to refine structure: *SHELXL97* (Sheldrick, 2008 ▶); molecular graphics: *Mercury* (Macrae *et al.*, 2006 ▶); software used to prepare material for publication: *WinGX* (Farrugia, 2012 ▶).

## Supplementary Material

Crystal structure: contains datablock(s) global, I. DOI: 10.1107/S1600536813033874/is5326sup1.cif

Structure factors: contains datablock(s) I. DOI: 10.1107/S1600536813033874/is5326Isup2.hkl

Supporting information file. DOI: 10.1107/S1600536813033874/is5326Isup3.cml

Additional supporting information:  crystallographic information; 3D view; checkCIF report

## Figures and Tables

**Table 1:** Hydrogen-bond geometry (Å, °) *Cg*2, *Cg*3, *Cg*5 and *Cg*6 are the centroids of the C1*A*–C6*A*, C7*A*–C12*A*, C1*B*–C6*B* and C7*B*–C12*B* phenyl rings, respectively.

*D*—H⋯*A*	*D*—H	H⋯*A*	*D*⋯*A*	*D*—H⋯*A*
C3*A*—H3*A*⋯*Cg*5^i^	0.95	2.76	3.553 (2)	141
C8*A*—H8*A*⋯*Cg*2^ii^	0.95	2.97	3.740 (2)	139
C15*B*—H15*B*⋯*Cg*2^iii^	0.95	2.60	3.484 (2)	156
C16*A*—H16*A*⋯*Cg*6^iv^	0.95	2.72	3.5725 (19)	150
C16*B*—H16*B*⋯*Cg*3^v^	0.95	2.80	3.659 (2)	151

Symmetry codes: (i) 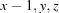; (ii) 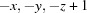; (iii) 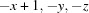; (iv) 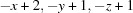; (v) 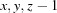.

**Fig. 1.**
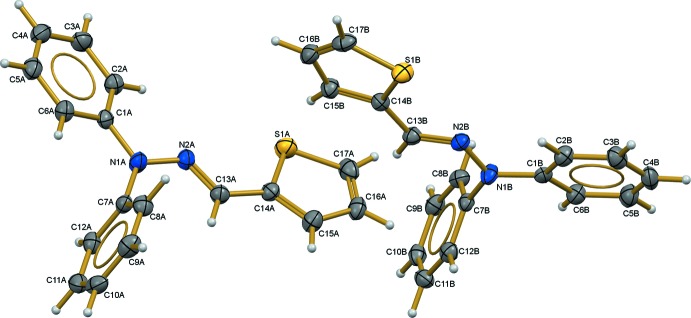
The molecular structure of the title compound. Displacement ellipsoids are drawn at the 60% probability level and H atoms are shown as circles of arbitrary size.

**Fig. 2.**
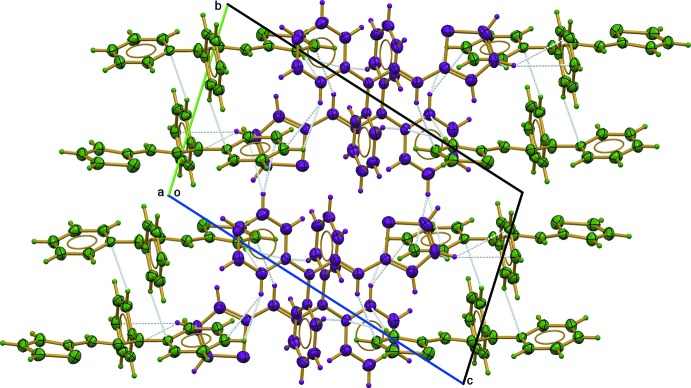
A packing diagram of the title compound viewed along the a axis. Intermolecular C—H···π interactions are indicated by dotted lines.

## supplementary crystallographic information

### 1. Comment

A great variety of hydrazine derivative compounds have been synthesized, which have been proven to control the growth of cancerous cells (Vogel *et al.*, 2008) or serve as antibiotics (Moreira *et al.*, 2012), inhibitors of HIV (Vicini *et al.*, 2009), anti-inflammatory, and antioxidant agents (Belkheiri *et al.*, 2010). Technologically speaking, hydrazone derivatives have been used to create materials with optical, electrochemical, photophysical and solar cells properties (Shen *et al.*, 2011). In this work we crystallized the (*E*)-1,1-diphenyl-2-(thiophen-2-ylmethylene)hydrazine.

In the title compound, the asymmetric unit consists of the two (*E*)-1,1-diphenyl-2-(thiophen-2-ylmethylene)hydrazine non-planar molecules (Fig. 1). Both molecules A (N1A to C17A/S1A) and B (N1B to C17B/S1B) show an *E* configuration with respect to the C═N double bond. The dihedral angle between the C1A–C6A and C7A–C12A rings is 89.32 (5)° for molecule A close proximity to the orthogonality (90°), while the angle between the C1B–C6B and C7B–C12B rings is 82.80 (5)° for molecule B. The (thiophen-2-ylmethylene)hydrazine group deviates from planarity with an r.m.s. deviation of fitted atoms of 0.0546 [equation: 9.774 (1) *x* - 2.803 (5) *y* - 5.122 (8) *z* = 9.501 (5)] and 0.0331 [equation: 2.970 (5) *x* - 9.686 (1) *y* + 5.981 (8) *z* = 6.359 (5)] for molecules A and B, respectively. The N2A—N1A—C1A [116.17 (13)°] and N2B—N1B—C1B [116.88 (13)°] angles are slightly shorter than the mean (120.28°) value with σ =1.19 in the Cambridge Structural Database, while C13A—N2A—N1A [119.40 (14)°] and C13B—N2B—N1B [118.81 (13)°] angles are slightly larger than the mean (116.14°) reported. In the crystal, C—H···π interactions (Table 1) link the molecule into a three-dimensional network (Fig. 2).

### 2. Experimental

491 mg (2.6 mmol) phenylhydrazine were dissolved in ethanol and acetic acid (0.5 ml) was slowly added into this solution while stirring, 300 mg (2.6 mmol) of thiophene-2-carbaldehyde, were added drop by drop into the above solution strongly stirring and the resulting mixture was kept at room temperature until it became a beige-coloured solution. After one and a half hours the solution precipitated. The reaction was monitored by TLC, aluminium AlugramSil G/UV254. The mixture was separated with filtration in *vacuo* system and the precipitate was washed three times with cold methanol. Recrystallization was performed with acetonitrile to obtain amber crystals for X-ray analysis. Yield 90%, amber needle, UV λ_max_ = 345.15 nm. FT IR (film): (cm^-1^): 3098 ν(C—H), 1586 ν(C=C—S), 1448, 1371 ν(C=N), 1292 ν(C—N), 854 γ(CH-thiophene). ^1^H NMR (400 MHz, (CD_3_)_2_CO: (δ/p.p.m.): 7.45(m,4*H*,C3'), 7.37 (t,1*H*,C3), 7.36(s,1*H*,Ci), 7.19 (m, 6H,C2',C4'), 7.02 (m, 1H,C5), 6.98 (dd, 1H,C4). ^13^C NMR (400 MHz, (CD_3_)_2_CO): (δ/p.p.m.): 141.67, 130.53, 129.89, 127.32, 126.99, 125.61, 124.64, 122.25. MS—EI: m/*z* = 278.37 *M*^+^. C_17_H_14_N_2_S.

### 3. Refinement

H atoms bonded to C atoms were placed in geometrical idealized positions and were refined as riding on their parent atoms, with C—H = 0.95 Å and with *U*_iso_(H) = 1.2*U*_eq_(C).

### Crystal data

**Table:**

C_17_H_14_N_2_S	*Z* = 4
*M**_r_* = 278.36	*F*(000) = 584
Triclinic, *P*1	*D*_x_ = 1.284 Mg m^−^^3^
Hall symbol: -P 1	Mo *K*α radiation, λ = 0.71073 Å
*a* = 9.8336 (11) Å	Cell parameters from 6444 reflections
*b* = 9.8665 (9) Å	θ = 3.6–26.0°
*c* = 16.6357 (8) Å	µ = 0.22 mm^−^^1^
α = 100.290 (6)°	*T* = 144 K
β = 101.696 (7)°	Prism, colourless
γ = 109.129 (9)°	0.58 × 0.51 × 0.36 mm
*V* = 1439.9 (2) Å^3^	

### Data collection

**Table:**

Agilent Xcalibur (Atlas, Gemini) diffractometer	5672 independent reflections
Graphite monochromator	4856 reflections with *I* > 2σ(*I*)
Detector resolution: 10.4685 pixels mm^-1^	*R*_int_ = 0.020
ω scans	θ_max_ = 26.1°, θ_min_ = 3.6°
Absorption correction: multi-scan (*CrysAlis RED*; Agilent, 2012)	*h* = −12→12
*T*_min_ = 0.916, *T*_max_ = 0.939	*k* = −10→12
10771 measured reflections	*l* = −20→20

### Refinement

**Table:**

Refinement on *F*^2^	0 restraints
Least-squares matrix: full	H-atom parameters constrained
*R*[*F*^2^ > 2σ(*F*^2^)] = 0.039	*w* = 1/[σ^2^(*F*_o_^2^) + (0.0382*P*)^2^ + 0.4189*P*] where *P* = (*F*_o_^2^ + 2*F*_c_^2^)/3
*wR*(*F*^2^) = 0.099	(Δ/σ)_max_ < 0.001
*S* = 1.07	Δρ_max_ = 0.23 e Å^−^^3^
5672 reflections	Δρ_min_ = −0.33 e Å^−^^3^
361 parameters	

### Special details

**Table:**

Geometry. All e.s.d.'s (except the e.s.d. in the dihedral angle between two l.s. planes) are estimated using the full covariance matrix. The cell e.s.d.'s are taken into account individually in the estimation of e.s.d.'s in distances, angles and torsion angles; correlations between e.s.d.'s in cell parameters are only used when they are defined by crystal symmetry. An approximate (isotropic) treatment of cell e.s.d.'s is used for estimating e.s.d.'s involving l.s. planes.

### Fractional atomic coordinates and isotropic or equivalent isotropic displacement parameters (Å^2^)

**Table:**

	*x*	*y*	*z*	*U*_iso_*/*U*_eq_	
S1A	0.45980 (5)	0.49688 (5)	0.64718 (3)	0.03525 (13)	
N1A	0.22725 (17)	0.04849 (15)	0.46812 (8)	0.0294 (3)	
N2A	0.29506 (15)	0.18305 (15)	0.52848 (8)	0.0258 (3)	
C1A	0.14928 (18)	0.05017 (18)	0.38736 (10)	0.0230 (3)	
C2A	0.17908 (19)	0.18184 (19)	0.36238 (11)	0.0277 (4)	
H2A	0.2535	0.272	0.3995	0.033*	
C3A	0.0996 (2)	0.1807 (2)	0.28310 (11)	0.0333 (4)	
H3A	0.1199	0.2709	0.2664	0.04*	
C4A	−0.0092 (2)	0.0502 (2)	0.22784 (11)	0.0346 (4)	
H4A	−0.0628	0.0505	0.1736	0.042*	
C5A	−0.0385 (2)	−0.0800 (2)	0.25262 (11)	0.0328 (4)	
H5A	−0.113	−0.1699	0.2152	0.039*	
C6A	0.03982 (19)	−0.08112 (19)	0.33190 (10)	0.0284 (4)	
H6A	0.0187	−0.1715	0.3483	0.034*	
C7A	0.21917 (18)	−0.08886 (17)	0.48812 (10)	0.0238 (3)	
C8A	0.11947 (19)	−0.15270 (19)	0.53117 (11)	0.0294 (4)	
H8A	0.0551	−0.106	0.5474	0.035*	
C9A	0.1137 (2)	−0.28481 (19)	0.55049 (11)	0.0329 (4)	
H9A	0.0454	−0.3287	0.5802	0.039*	
C10A	0.2069 (2)	−0.35305 (19)	0.52681 (11)	0.0328 (4)	
H10A	0.2022	−0.444	0.5399	0.039*	
C11A	0.30686 (19)	−0.28940 (19)	0.48412 (11)	0.0320 (4)	
H11A	0.3709	−0.3366	0.4679	0.038*	
C12A	0.31402 (18)	−0.15642 (18)	0.46491 (10)	0.0274 (4)	
H12A	0.3835	−0.1119	0.436	0.033*	
C13A	0.35204 (18)	0.18705 (18)	0.60598 (10)	0.0259 (4)	
H13A	0.3475	0.0974	0.6209	0.031*	
C14A	0.42304 (18)	0.32729 (18)	0.67056 (10)	0.0250 (4)	
C15A	0.46818 (19)	0.34694 (19)	0.75658 (11)	0.0309 (4)	
H15A	0.4591	0.267	0.7823	0.037*	
C16A	0.52983 (18)	0.49809 (19)	0.80343 (11)	0.0309 (4)	
H16A	0.5649	0.5303	0.8638	0.037*	
C17A	0.5333 (2)	0.5912 (2)	0.75320 (12)	0.0347 (4)	
H17A	0.5718	0.6965	0.7738	0.042*	
S1B	0.49868 (5)	0.03271 (5)	−0.12555 (3)	0.03013 (12)	
N1B	0.96060 (14)	0.28458 (16)	0.04326 (8)	0.0260 (3)	
N2B	0.82065 (14)	0.20862 (14)	−0.01346 (8)	0.0233 (3)	
C1B	0.96712 (18)	0.36467 (17)	0.12427 (10)	0.0221 (3)	
C2B	0.84018 (19)	0.33497 (19)	0.15375 (11)	0.0271 (4)	
H2B	0.7471	0.2606	0.1192	0.032*	
C3B	0.8508 (2)	0.4147 (2)	0.23387 (11)	0.0320 (4)	
H3B	0.764	0.3944	0.2537	0.038*	
C4B	0.9854 (2)	0.52359 (19)	0.28560 (11)	0.0331 (4)	
H4B	0.9919	0.576	0.3409	0.04*	
C5B	1.1098 (2)	0.55446 (19)	0.25521 (11)	0.0331 (4)	
H5B	1.2022	0.6302	0.2896	0.04*	
C6B	1.10198 (19)	0.47654 (18)	0.17521 (11)	0.0273 (4)	
H6B	1.1884	0.4993	0.1551	0.033*	
C7B	1.09374 (17)	0.29233 (17)	0.01841 (10)	0.0217 (3)	
C8B	1.15424 (18)	0.39775 (19)	−0.02261 (10)	0.0270 (4)	
H8B	1.1103	0.4682	−0.0321	0.032*	
C9B	1.27897 (19)	0.40038 (19)	−0.04985 (10)	0.0284 (4)	
H9B	1.3209	0.4731	−0.0777	0.034*	
C10B	1.34251 (18)	0.29745 (19)	−0.03651 (10)	0.0274 (4)	
H10B	1.4278	0.2992	−0.0555	0.033*	
C11B	1.28180 (19)	0.19156 (19)	0.00450 (11)	0.0292 (4)	
H11B	1.3255	0.1209	0.0137	0.035*	
C12B	1.15722 (18)	0.18903 (18)	0.03205 (10)	0.0254 (4)	
H12B	1.1155	0.1167	0.0602	0.03*	
C13B	0.81062 (18)	0.14782 (17)	−0.09080 (10)	0.0235 (3)	
H13B	0.8991	0.1555	−0.1075	0.028*	
C14B	0.66548 (17)	0.06796 (17)	−0.15229 (10)	0.0226 (3)	
C15B	0.63864 (19)	0.01036 (19)	−0.23772 (11)	0.0279 (4)	
H15B	0.7159	0.0176	−0.2649	0.033*	
C16B	0.48434 (19)	−0.06090 (19)	−0.28138 (11)	0.0304 (4)	
H16B	0.447	−0.1064	−0.3409	0.036*	
C17B	0.39578 (19)	−0.05744 (19)	−0.22952 (11)	0.0313 (4)	
H17B	0.2893	−0.0997	−0.2482	0.038*	

### Atomic displacement parameters (Å^2^)

**Table:**

	*U*^11^	*U*^22^	*U*^33^	*U*^12^	*U*^13^	*U*^23^
S1A	0.0430 (3)	0.0264 (2)	0.0326 (3)	0.0123 (2)	0.0031 (2)	0.00904 (19)
N1A	0.0417 (9)	0.0208 (7)	0.0206 (7)	0.0096 (6)	0.0033 (7)	0.0038 (6)
N2A	0.0285 (7)	0.0226 (7)	0.0232 (7)	0.0079 (6)	0.0062 (6)	0.0031 (6)
C1A	0.0251 (8)	0.0267 (8)	0.0198 (8)	0.0115 (7)	0.0092 (7)	0.0055 (7)
C2A	0.0304 (9)	0.0260 (9)	0.0263 (9)	0.0095 (7)	0.0091 (8)	0.0068 (7)
C3A	0.0431 (11)	0.0354 (10)	0.0301 (9)	0.0210 (9)	0.0133 (9)	0.0145 (8)
C4A	0.0360 (10)	0.0478 (11)	0.0251 (9)	0.0230 (9)	0.0065 (8)	0.0104 (8)
C5A	0.0287 (9)	0.0366 (10)	0.0274 (9)	0.0113 (8)	0.0042 (8)	0.0009 (8)
C6A	0.0301 (9)	0.0266 (9)	0.0258 (9)	0.0085 (7)	0.0078 (8)	0.0047 (7)
C7A	0.0285 (9)	0.0218 (8)	0.0183 (8)	0.0083 (7)	0.0033 (7)	0.0044 (6)
C8A	0.0260 (9)	0.0316 (9)	0.0295 (9)	0.0102 (7)	0.0087 (8)	0.0057 (8)
C9A	0.0322 (9)	0.0311 (10)	0.0304 (9)	0.0040 (8)	0.0081 (8)	0.0125 (8)
C10A	0.0337 (10)	0.0227 (9)	0.0324 (10)	0.0072 (8)	−0.0050 (8)	0.0074 (8)
C11A	0.0297 (9)	0.0309 (9)	0.0318 (10)	0.0154 (8)	0.0012 (8)	0.0007 (8)
C12A	0.0247 (8)	0.0282 (9)	0.0235 (8)	0.0057 (7)	0.0063 (7)	0.0013 (7)
C13A	0.0291 (9)	0.0240 (8)	0.0249 (9)	0.0101 (7)	0.0074 (7)	0.0071 (7)
C14A	0.0228 (8)	0.0250 (8)	0.0250 (8)	0.0082 (7)	0.0047 (7)	0.0053 (7)
C15A	0.0325 (9)	0.0296 (9)	0.0258 (9)	0.0073 (8)	0.0052 (8)	0.0075 (7)
C16A	0.0252 (9)	0.0345 (10)	0.0238 (9)	0.0065 (8)	0.0032 (7)	−0.0014 (8)
C17A	0.0293 (9)	0.0258 (9)	0.0401 (11)	0.0083 (8)	0.0037 (8)	−0.0017 (8)
S1B	0.0233 (2)	0.0401 (3)	0.0284 (2)	0.01185 (19)	0.00921 (19)	0.0106 (2)
N1B	0.0180 (7)	0.0343 (8)	0.0204 (7)	0.0075 (6)	0.0031 (6)	0.0014 (6)
N2B	0.0205 (7)	0.0243 (7)	0.0221 (7)	0.0069 (6)	0.0031 (6)	0.0050 (6)
C1B	0.0262 (8)	0.0232 (8)	0.0187 (8)	0.0114 (7)	0.0047 (7)	0.0077 (7)
C2B	0.0258 (9)	0.0286 (9)	0.0258 (9)	0.0079 (7)	0.0082 (7)	0.0082 (7)
C3B	0.0393 (10)	0.0343 (10)	0.0314 (9)	0.0176 (8)	0.0194 (9)	0.0129 (8)
C4B	0.0491 (11)	0.0275 (9)	0.0254 (9)	0.0173 (8)	0.0138 (9)	0.0044 (7)
C5B	0.0373 (10)	0.0269 (9)	0.0268 (9)	0.0084 (8)	0.0030 (8)	0.0015 (7)
C6B	0.0268 (9)	0.0284 (9)	0.0253 (9)	0.0097 (7)	0.0061 (7)	0.0063 (7)
C7B	0.0184 (8)	0.0244 (8)	0.0174 (7)	0.0058 (6)	0.0022 (6)	0.0012 (6)
C8B	0.0266 (9)	0.0284 (9)	0.0267 (9)	0.0117 (7)	0.0050 (7)	0.0097 (7)
C9B	0.0290 (9)	0.0293 (9)	0.0242 (9)	0.0064 (7)	0.0078 (7)	0.0094 (7)
C10B	0.0217 (8)	0.0328 (9)	0.0240 (8)	0.0084 (7)	0.0078 (7)	0.0009 (7)
C11B	0.0278 (9)	0.0258 (9)	0.0329 (9)	0.0126 (7)	0.0053 (8)	0.0039 (7)
C12B	0.0262 (9)	0.0218 (8)	0.0248 (8)	0.0062 (7)	0.0056 (7)	0.0056 (7)
C13B	0.0218 (8)	0.0253 (8)	0.0234 (8)	0.0089 (7)	0.0060 (7)	0.0065 (7)
C14B	0.0230 (8)	0.0226 (8)	0.0232 (8)	0.0094 (7)	0.0073 (7)	0.0060 (7)
C15B	0.0250 (9)	0.0321 (9)	0.0254 (9)	0.0111 (7)	0.0073 (7)	0.0044 (7)
C16B	0.0302 (9)	0.0284 (9)	0.0252 (9)	0.0092 (7)	−0.0012 (8)	0.0033 (7)
C17B	0.0217 (8)	0.0317 (9)	0.0356 (10)	0.0072 (7)	0.0004 (8)	0.0113 (8)

### Geometric parameters (Å, º)

**Table:**

S1A—C17A	1.7190 (19)	S1B—C17B	1.7198 (18)
S1A—C14A	1.7247 (17)	S1B—C14B	1.7281 (16)
N1A—N2A	1.3739 (19)	N1B—N2B	1.3751 (18)
N1A—C1A	1.411 (2)	N1B—C1B	1.413 (2)
N1A—C7A	1.433 (2)	N1B—C7B	1.434 (2)
N2A—C13A	1.285 (2)	N2B—C13B	1.288 (2)
C1A—C2A	1.392 (2)	C1B—C2B	1.394 (2)
C1A—C6A	1.394 (2)	C1B—C6B	1.395 (2)
C2A—C3A	1.386 (2)	C2B—C3B	1.386 (2)
C2A—H2A	0.95	C2B—H2B	0.95
C3A—C4A	1.387 (3)	C3B—C4B	1.387 (3)
C3A—H3A	0.95	C3B—H3B	0.95
C4A—C5A	1.378 (3)	C4B—C5B	1.381 (3)
C4A—H4A	0.95	C4B—H4B	0.95
C5A—C6A	1.390 (2)	C5B—C6B	1.386 (2)
C5A—H5A	0.95	C5B—H5B	0.95
C6A—H6A	0.95	C6B—H6B	0.95
C7A—C8A	1.383 (2)	C7B—C8B	1.384 (2)
C7A—C12A	1.387 (2)	C7B—C12B	1.386 (2)
C8A—C9A	1.384 (2)	C8B—C9B	1.385 (2)
C8A—H8A	0.95	C8B—H8B	0.95
C9A—C10A	1.379 (3)	C9B—C10B	1.382 (2)
C9A—H9A	0.95	C9B—H9B	0.95
C10A—C11A	1.379 (3)	C10B—C11B	1.387 (2)
C10A—H10A	0.95	C10B—H10B	0.95
C11A—C12A	1.388 (2)	C11B—C12B	1.387 (2)
C11A—H11A	0.95	C11B—H11B	0.95
C12A—H12A	0.95	C12B—H12B	0.95
C13A—C14A	1.445 (2)	C13B—C14B	1.445 (2)
C13A—H13A	0.95	C13B—H13B	0.95
C14A—C15A	1.367 (2)	C14B—C15B	1.369 (2)
C15A—C16A	1.414 (2)	C15B—C16B	1.413 (2)
C15A—H15A	0.95	C15B—H15B	0.95
C16A—C17A	1.345 (3)	C16B—C17B	1.348 (3)
C16A—H16A	0.95	C16B—H16B	0.95
C17A—H17A	0.95	C17B—H17B	0.95
			
C17A—S1A—C14A	91.75 (9)	C17B—S1B—C14B	91.65 (8)
N2A—N1A—C1A	116.17 (13)	N2B—N1B—C1B	116.88 (13)
N2A—N1A—C7A	122.29 (13)	N2B—N1B—C7B	121.16 (12)
C1A—N1A—C7A	121.09 (13)	C1B—N1B—C7B	121.74 (13)
C13A—N2A—N1A	119.40 (14)	C13B—N2B—N1B	118.81 (13)
C2A—C1A—C6A	119.30 (15)	C2B—C1B—C6B	119.38 (15)
C2A—C1A—N1A	120.86 (15)	C2B—C1B—N1B	121.00 (15)
C6A—C1A—N1A	119.83 (15)	C6B—C1B—N1B	119.62 (14)
C3A—C2A—C1A	119.70 (16)	C3B—C2B—C1B	119.53 (16)
C3A—C2A—H2A	120.1	C3B—C2B—H2B	120.2
C1A—C2A—H2A	120.1	C1B—C2B—H2B	120.2
C2A—C3A—C4A	121.05 (17)	C2B—C3B—C4B	121.33 (16)
C2A—C3A—H3A	119.5	C2B—C3B—H3B	119.3
C4A—C3A—H3A	119.5	C4B—C3B—H3B	119.3
C5A—C4A—C3A	119.19 (16)	C5B—C4B—C3B	118.76 (16)
C5A—C4A—H4A	120.4	C5B—C4B—H4B	120.6
C3A—C4A—H4A	120.4	C3B—C4B—H4B	120.6
C4A—C5A—C6A	120.59 (17)	C4B—C5B—C6B	120.98 (17)
C4A—C5A—H5A	119.7	C4B—C5B—H5B	119.5
C6A—C5A—H5A	119.7	C6B—C5B—H5B	119.5
C5A—C6A—C1A	120.16 (16)	C5B—C6B—C1B	119.99 (16)
C5A—C6A—H6A	119.9	C5B—C6B—H6B	120
C1A—C6A—H6A	119.9	C1B—C6B—H6B	120
C8A—C7A—C12A	120.14 (15)	C8B—C7B—C12B	120.21 (15)
C8A—C7A—N1A	120.52 (15)	C8B—C7B—N1B	120.15 (14)
C12A—C7A—N1A	119.34 (14)	C12B—C7B—N1B	119.57 (14)
C7A—C8A—C9A	119.80 (16)	C7B—C8B—C9B	119.86 (15)
C7A—C8A—H8A	120.1	C7B—C8B—H8B	120.1
C9A—C8A—H8A	120.1	C9B—C8B—H8B	120.1
C10A—C9A—C8A	120.23 (16)	C10B—C9B—C8B	120.13 (15)
C10A—C9A—H9A	119.9	C10B—C9B—H9B	119.9
C8A—C9A—H9A	119.9	C8B—C9B—H9B	119.9
C11A—C10A—C9A	120.12 (16)	C9B—C10B—C11B	120.04 (15)
C11A—C10A—H10A	119.9	C9B—C10B—H10B	120
C9A—C10A—H10A	119.9	C11B—C10B—H10B	120
C10A—C11A—C12A	120.06 (16)	C12B—C11B—C10B	119.91 (16)
C10A—C11A—H11A	120	C12B—C11B—H11B	120
C12A—C11A—H11A	120	C10B—C11B—H11B	120
C7A—C12A—C11A	119.65 (15)	C7B—C12B—C11B	119.83 (15)
C7A—C12A—H12A	120.2	C7B—C12B—H12B	120.1
C11A—C12A—H12A	120.2	C11B—C12B—H12B	120.1
N2A—C13A—C14A	120.29 (15)	N2B—C13B—C14B	120.34 (15)
N2A—C13A—H13A	119.9	N2B—C13B—H13B	119.8
C14A—C13A—H13A	119.9	C14B—C13B—H13B	119.8
C15A—C14A—C13A	126.66 (16)	C15B—C14B—C13B	126.43 (15)
C15A—C14A—S1A	110.41 (13)	C15B—C14B—S1B	110.52 (12)
C13A—C14A—S1A	122.92 (13)	C13B—C14B—S1B	123.05 (12)
C14A—C15A—C16A	113.27 (16)	C14B—C15B—C16B	113.16 (15)
C14A—C15A—H15A	123.4	C14B—C15B—H15B	123.4
C16A—C15A—H15A	123.4	C16B—C15B—H15B	123.4
C17A—C16A—C15A	112.64 (16)	C17B—C16B—C15B	112.77 (16)
C17A—C16A—H16A	123.7	C17B—C16B—H16B	123.6
C15A—C16A—H16A	123.7	C15B—C16B—H16B	123.6
C16A—C17A—S1A	111.92 (13)	C16B—C17B—S1B	111.90 (13)
C16A—C17A—H17A	124	C16B—C17B—H17B	124.1
S1A—C17A—H17A	124	S1B—C17B—H17B	124.1
			
C1A—N1A—N2A—C13A	171.50 (14)	C1B—N1B—N2B—C13B	172.48 (14)
C7A—N1A—N2A—C13A	−0.8 (2)	C7B—N1B—N2B—C13B	−2.2 (2)
N2A—N1A—C1A—C2A	19.8 (2)	N2B—N1B—C1B—C2B	17.7 (2)
C7A—N1A—C1A—C2A	−167.71 (15)	C7B—N1B—C1B—C2B	−167.66 (14)
N2A—N1A—C1A—C6A	−159.52 (14)	N2B—N1B—C1B—C6B	−161.46 (14)
C7A—N1A—C1A—C6A	12.9 (2)	C7B—N1B—C1B—C6B	13.2 (2)
C6A—C1A—C2A—C3A	0.2 (2)	C6B—C1B—C2B—C3B	−1.4 (2)
N1A—C1A—C2A—C3A	−179.22 (15)	N1B—C1B—C2B—C3B	179.43 (15)
C1A—C2A—C3A—C4A	−0.2 (3)	C1B—C2B—C3B—C4B	−0.1 (3)
C2A—C3A—C4A—C5A	0.2 (3)	C2B—C3B—C4B—C5B	1.5 (3)
C3A—C4A—C5A—C6A	−0.1 (3)	C3B—C4B—C5B—C6B	−1.3 (3)
C4A—C5A—C6A—C1A	0.0 (3)	C4B—C5B—C6B—C1B	−0.3 (3)
C2A—C1A—C6A—C5A	0.0 (2)	C2B—C1B—C6B—C5B	1.6 (2)
N1A—C1A—C6A—C5A	179.33 (15)	N1B—C1B—C6B—C5B	−179.22 (15)
N2A—N1A—C7A—C8A	75.1 (2)	N2B—N1B—C7B—C8B	83.36 (19)
C1A—N1A—C7A—C8A	−96.82 (19)	C1B—N1B—C7B—C8B	−91.07 (19)
N2A—N1A—C7A—C12A	−103.95 (18)	N2B—N1B—C7B—C12B	−93.54 (18)
C1A—N1A—C7A—C12A	84.1 (2)	C1B—N1B—C7B—C12B	92.03 (19)
C12A—C7A—C8A—C9A	−0.5 (2)	C12B—C7B—C8B—C9B	−0.2 (2)
N1A—C7A—C8A—C9A	−179.57 (15)	N1B—C7B—C8B—C9B	−177.12 (14)
C7A—C8A—C9A—C10A	−0.1 (3)	C7B—C8B—C9B—C10B	0.3 (2)
C8A—C9A—C10A—C11A	0.4 (3)	C8B—C9B—C10B—C11B	−0.3 (3)
C9A—C10A—C11A—C12A	0.0 (3)	C9B—C10B—C11B—C12B	0.0 (3)
C8A—C7A—C12A—C11A	0.9 (2)	C8B—C7B—C12B—C11B	0.0 (2)
N1A—C7A—C12A—C11A	179.96 (15)	N1B—C7B—C12B—C11B	176.93 (14)
C10A—C11A—C12A—C7A	−0.6 (2)	C10B—C11B—C12B—C7B	0.1 (2)
N1A—N2A—C13A—C14A	−179.61 (14)	N1B—N2B—C13B—C14B	−179.59 (13)
N2A—C13A—C14A—C15A	169.63 (17)	N2B—C13B—C14B—C15B	173.46 (16)
N2A—C13A—C14A—S1A	−9.3 (2)	N2B—C13B—C14B—S1B	−6.5 (2)
C17A—S1A—C14A—C15A	−0.57 (14)	C17B—S1B—C14B—C15B	−0.43 (13)
C17A—S1A—C14A—C13A	178.54 (15)	C17B—S1B—C14B—C13B	179.51 (14)
C13A—C14A—C15A—C16A	−178.07 (16)	C13B—C14B—C15B—C16B	−179.54 (15)
S1A—C14A—C15A—C16A	1.0 (2)	S1B—C14B—C15B—C16B	0.40 (19)
C14A—C15A—C16A—C17A	−1.0 (2)	C14B—C15B—C16B—C17B	−0.1 (2)
C15A—C16A—C17A—S1A	0.6 (2)	C15B—C16B—C17B—S1B	−0.2 (2)
C14A—S1A—C17A—C16A	0.00 (14)	C14B—S1B—C17B—C16B	0.35 (14)

### Hydrogen-bond geometry (Å, º)

*Cg*2, *Cg*3, *Cg*5 and *Cg*6 are the centroids of the C1A–C6A, C7A–C12A, C1B–C6B and C7B–C12B phenyl rings, respectively.

**Table:**

*D*—H···*A*	*D*—H	H···*A*	*D*···*A*	*D*—H···*A*
C3*A*—H3*A*···*Cg*5^i^	0.95	2.76	3.553 (2)	141
C8*A*—H8*A*···*Cg*2^ii^	0.95	2.97	3.740 (2)	139
C15*B*—H15*B*···*Cg*2^iii^	0.95	2.60	3.484 (2)	156
C16*A*—H16*A*···*Cg*6^iv^	0.95	2.72	3.5725 (19)	150
C16*B*—H16*B*···*Cg*3^v^	0.95	2.80	3.659 (2)	151

Symmetry codes: (i) *x*−1, *y*, *z*; (ii) −*x*, −*y*, −*z*+1; (iii) −*x*+1, −*y*, −*z*; (iv) −*x*+2, −*y*+1, −*z*+1; (v) *x*, *y*, *z*−1.
